# Neuropsychological Consequences of Massive Trauma: Implications and Clinical Interventions

**DOI:** 10.3390/medicina59122128

**Published:** 2023-12-06

**Authors:** Maria Theodoratou, Georgios A. Kougioumtzis, Vasiliki Yotsidi, Maria Sofologi, Dimitra Katsarou, Kalliopi Megari

**Affiliations:** 1 Department of Psychology, School of Health Sciences, Neapolis University Pafos, Paphos 8042, Cyprus; gkougioum@ppp.uoa.gr (G.A.K.); m.sofologi@uoi.gr (M.S.); d.katsarou@aegean.gr (D.K.); 2 Department of Social Sciences, Hellenic Open University, 263 35 Patras, Greece; v.yiotsidi@panteion.gr; 3 Department of Turkish Studies and Modern Asian Studies, Faculty of Economic and Political Sciences, National and Kapodistrian University of Athens, 157 72 Athens, Greece; 4 Department of Psychology, Panteion University of Social and Political Sciences, 157 72 Athens, Greece; 5 Laboratory of Psychology, Department of Early Childhood Education, School of Education, University of Ioannina, 451 10 Ioannina, Greece; 6 Institute of Humanities and Social Sciences, University Research Centre of Ioannina (U.R.C.I.), 451 10 Ioannina, Greece; 7 Department of Preschool Education Sciences and Educational Design, Faculty of Humanities, University of the Aegean, 811 00 Mytilene, Greece; 8 City College, University of York, Europe Campus, 546 26 Thessaloniki, Greece; kmegari@psy.auth.gr; 9 Department of Psychology, School of Social Sciences, UOWM, 531 00 Florina, Greece; 10 School of Psychology, Aristotle University of Thessaloniki, 541 24 Thessaloniki, Greece

**Keywords:** massive trauma, neuropsychology, neuroplasticity, clinical intervention, cognitive rehabilitation, therapeutic interventions

## Abstract

Traumatic events, especially massive trauma resulting from catastrophic incidents, wars, or severe abuse can elicit significant neuropsychological alterations, with profound implications for cognitive, emotional, and behavioral functioning. This mini-review delineates the primary neural changes post-trauma and underscores the importance of timely neuropsychological and clinical interventions. Specific brain regions, including the amygdala and prefrontal cortex, undergo physiological changes that can lead to memory impairments, attention deficits, and emotional disturbances. PTSD, a commonly diagnosed condition post-trauma, exemplifies the intricate relationship between trauma and memory processing. Furthermore, the concept of neuroplasticity, the brain’s inherent ability to adapt and rewire, offers hope for recovery. Current clinical interventions, such as cognitive behavioral therapy, mindfulness practices, and biofeedback, leverage this neuroplastic potential to foster healing. The review underscores the vital importance of early intervention to mitigate long-term neuropsychological impacts, emphasizing the role of timely and targeted clinical interventions. The synthesis of this knowledge is crucial for clinicians, allowing for informed therapeutic approaches that holistically address both the physiological and psychological dimensions of trauma.

## 1. Introduction

Research on massive traumas, such as natural disasters, wars, or pandemics, is particularly relevant in today’s world [1]. These large-scale events have far-reaching implications, impacting entire communities and populations [2]. A thorough understanding of the neuropsychological consequences of such trauma is critical for guiding public health interventions and informing policy decisions. The effects of major traumatic events extend beyond psychological distress to include profound neuropsychological changes that can affect critical brain regions responsible for memory, emotion regulation, and threat detection [3]. This mini-review aims to elucidate these neuropsychological changes and advocate for specific, evidence-based interventions. In this context, neuropsychology offers insights into the complex relationship between brain functioning and our behavior [4]. When individuals experience significant traumatic events—whether natural disasters, wartime experiences, intense personal abuse, or other deeply distressing circumstances—the neuropsychological impact can be significant, potentially leading to altered behavior and cognitive processing [5].

It is important to note that the current understanding of these consequences may be limited due to the variability in trauma response, the challenges in conducting longitudinal research, and the heterogeneity of the traumatic events themselves. All these factors complicate understanding and treating neuropsychological effects. Thus, the current literature may not fully address the range of individual experiences or long-term neuropsychological effects resulting from trauma. This review acknowledges these limitations and emphasizes the importance of continued research efforts to improve our understanding of the complex neuropsychological consequences of trauma [6].

## 2. Methods

The narrative review takes a comprehensive approach to capturing the range of neuropsychological outcomes resulting from massive trauma and the effectiveness of interventions aimed at addressing these outcomes. The literature search was conducted in multiple databases, including PubMed, PsycINFO, Scopus, Embase, Ebsco, Google Scholar, and CINAHL, and included a targeted set of keywords: “Massive trauma”, “neuropsychological consequences”, “neuropsychological interventions”, and “clinical interventions”, alongside “cognitive behavioral therapy” and “neuroplasticity.” Selection criteria were carefully developed to include English-language studies comprehensively examining neuropsychological consequences of trauma and interventions to address these consequences, with a publishing time between 1990 and 2023 to ensure the inclusion of the most current and relevant data. During the screening process, titles and abstracts directly related to the neuropsychological sequelae of trauma and subsequent interventions were identified. An extensive evaluation of full-text articles was then conducted, examining study designs, outcome measures, and the strength of evidence provided regarding the efficacy of neuropsychological and clinical interventions. Articles were sought that provided examples of effective interventions, such as those describing the use and outcomes of cognitive behavioral strategies to improve post-traumatic cognitive dysfunction or the use of neurorehabilitation techniques to promote neuroplasticity and functional recovery. Our review included studies evaluating the efficacy of multidisciplinary approaches, including psychotherapeutic and pharmacological treatments.

The literature we reviewed encompassed randomized controlled trials assessing specific therapeutic interventions, as well as longitudinal studies tracking neuropsychological recovery over time. The objective of this study is to comprehensively examine the neuropsychological consequences of massive trauma and the effectiveness of various interventions in clinical practice. This research aims to contribute towards an informed understanding of the best practices for trauma care.

## 3. Results

What is massive trauma?

Massive or mass trauma, while lacking a single operational definition due to the diverse nature of traumatic events, can be considered from several perspectives. It could be defined quantitatively, for example, by the sheer number of victims of catastrophic events such as earthquakes, wars, or major terrorist attacks [6,7]. Alternatively, mass trauma might refer to persistent and widespread traumatic conditions, such as those found in high-crime neighborhoods or refugee or concentration camps. Unprecedented events, such as biochemical weapons use, can cause widespread panic beyond immediate harm. Common to these diverse events is a marked mismatch between needs and resources, and between trauma and the potential to heal. Essentially, mass trauma occurs when adaptive strategies fail and vulnerabilities emerge, at least temporarily. The following sections outline some of the key neuropsychological aspects.

### 3.1. Cultural and Individual Differences with Response to Trauma

Throughout history, discrimination based on race or ethnicity has deeply scarred humanity. Genocidal campaigns aimed at eliminating specific racial, ethnic, or cultural groups have caused untold suffering and loss of life around the world. The term “historical trauma” is used to describe the lasting effects of such genocides, which affect not only the direct victims but also generations of their descendants [8]. Memories of such catastrophic events often result in a state of perpetual vigilance within affected communities, serving as a safeguard against the potential recurrence of such violence. This vigilance can be adaptive, fostering a willingness to combat future threats of discrimination or violence. However, it can also lead to a persistent state of heightened awareness, like that seen in PTSD, which can interfere with daily life and affect overall well-being.

The study of massive trauma and PTSD must be sensitive to cultural and individual differences in the perception and management of trauma [9]. Reactions to trauma are not uniform and depend on numerous factors, including cultural background, personal history, and social support systems. When studying the impact of trauma on ethnoracial groups and individuals with a history of historical trauma, it is critical to consider these variations. This approach highlights the significance of culturally competent understanding, which recognizes that the ways in which trauma is coped with and the meanings ascribed to trauma are diverse and deeply personal.

### 3.2. Neuropsychological Consequences of Massive Trauma

Massive trauma, especially when resulting in post-traumatic stress disorder (PTSD), has a number of profound neuropsychological consequences [10,11]. A prominent concern in these populations is apparent cognitive impairment, particularly in attention and working memory. These cognitive domains, fundamental to efficient executive functioning, appear to be impaired, creating functional and behavioral challenges in daily task performance and decision-making [12,13,14]. Furthermore, executive functioning, including critical skills such as mental agility and cognitive control, is impaired in PTSD patients. Such impairments make it difficult to adapt to changing circumstances and to control one’s cognitive processes [15,16,17]. Another major effect of massive trauma is the reduction in information processing speed. These neuropsychological effects reveal the pervasive and debilitating nature of massive trauma, illustrating how its effects are not exclusively emotional and psychological, but rather spill over into cognitive domains, complicating recovery and everyday adaptability [18]. Specifically, the following consequences are observed.

### 3.3. Neurological Alterations Following Traumatic Experiences

Massive trauma may result in physiological changes in the brain, particularly in areas involved in memory, emotion regulation, and threat recognition [19]. It also affects areas related to impulsivity and decision-making. In particular, the amygdala, a key region for detecting danger and producing fear responses, may show increased activity [20]. A growing body of evidence suggests that PTSD may be understood, in part, as dysregulation of fear perception [21,22]. This perspective is particularly relevant due to the critical role of the amygdala, linked to fear responses and interacting with the hippocampus and medial prefrontal cortex [23]. Conversely, the prefrontal cortex, which is involved in decision-making and impulse control, may show reduced activity, leading to difficulties in emotion regulation [24,25]. Specifically, this brain region, along with areas such as the basal ganglia, is critical for the regulation of impulsive acts. One reason for this is that trauma may interfere with typical prefrontal cortical functioning [26]. When trauma disrupts normal prefrontal cortex functioning, it can create an imbalance that prioritizes speed over accuracy, leading to increased impulsivity [27]. In addition, dysfunction in the brain’s dorsal prefrontal networks may be associated with these cognitive challenges. Such deficits may influence the overall clinical presentation of PTSD and result in the use of coping mechanisms such as avoidance [28].

### 3.4. Memory Impairments

Major traumatic events can have a marked impact on the processing of memories. This is exemplified by conditions such as PTSD, which can cause individuals to experience vivid memories [29]. In addition, a traumatic event can lead to the emergence of intrusive memories, especially shortly after the trauma [30]. These memories can be distressing and interfere with daily functioning, although they often diminish over time [31,32,33,34] However, they can also serve adaptive purposes, such as warning signals or assisting autobiographical memory coherence [35,36]. Dealing with these memories is critical to trauma prevention and early intervention strategies. Alternatively, people may have trouble recalling the event [28].

### 3.5. Emotional Consequences

Emotional symptoms may include depressive symptoms, generalized anxiety, and heightened emotional reactivity [37]. An important aspect of understanding post-traumatic stress reactions is the challenge of emotion regulation [38]. Individuals with PTSD often struggle with managing their negative emotions, which is essential for coping with the intense emotional reactions towards trauma reminders [38,39]. Evidence highlights that non-acceptance of negative emotions can exacerbate trauma-related distress, leading to secondary emotions such as guilt or shame [40,41,42]. Besides, it may also divert resources away from adaptive behaviors that enhance overall well-being. In addition, difficulties in emotion regulation are associated with avoidance strategies [43,44]. For instance, problems in identifying and describing emotions after trauma, known as secondary alexithymia, may act as a form of avoidance, further exacerbating post-traumatic stress reactions. Overall, emotion regulation challenges appear to play a central role in the maintenance of post-traumatic symptoms.

### 3.6. Neuropsychological and Clinical Interventions

Neuropsychological interventions after trauma can take advantage of the brain’s inherent ability to adapt and change, known as neuroplasticity [45], by using therapeutic techniques to promote healing and rewire maladaptive brain patterns [46]. Such interventions may include cognitive behavioral therapy, mindfulness practices, and even biofeedback to help trauma survivors regain control and improve cognitive functioning [47]. Early neuropsychological and clinical interventions after a traumatic event can provide immediate support and therapy, potentially mitigating long-term neuropsychological effects of trauma, helping the brain establish healthier patterns, and reducing the risk for chronic conditions like PTSD [39,41].

Individuals affected by trauma should be supported in a manner that is respectful, patient, and understanding; a safe and secure environment in which one’s feelings are acknowledged and validated should be provided [42]; active, non-judgmental listening should be employed, allowing for the uninhibited expression of experiences and emotions. Trauma survivors are reassured that emotional responses such as fear, detachment, or anxiety are natural after traumatic events [43] and are gently encouraged to seek professional help when they feel ready, as trauma specialists can provide the appropriate care. It is also important for helpers to remember the importance of self-care, as the maintenance of their own mental well-being is essential to the effective provision of help to others.

Neuropsychological interventions aim to address the cognitive, emotional, and behavioral outcomes of trauma by drawing on the brain’s adaptive and transformative potential. The following targeted clinical interventions can be used to address the neuropsychological effects of massive trauma.

### 3.7. Cognitive Rehabilitation

Effective cognitive rehabilitation often involves a combination of strategic approaches. This includes goal setting, where tasks are broken down into manageable increments. It has been suggested that these methods can lead to improved outcomes in rehabilitating cognitive functions [33]. Prioritization can help people manage their daily tasks more efficiently and reduce cognitive load.

In addition, memory, which is often impaired following a traumatic event, can be improved through various enhancement techniques, and mnemonic approaches have been widely recognized for their effectiveness in improving retrieval [34,36]. In today’s digital age, electronic reminders on devices like smartphones can provide comfort. However, for those who prefer a more tangible approach, traditional note-taking or jotting down reminders can serve as a valuable tool for strengthening memory.

Advanced therapeutic interventions, such as bio- and neuro-feedback, provide individuals with real-time insights into their physiological responses, enabling them to gain a comprehensive understanding of their body’s reactions [47]. Neurofeedback, for example, focuses on specific brainwave dynamics aiming to correct patterns associated with traumatic experiences, paving the way for full recovery.

### 3.8. Cognitive Behavioral Therapies (CBTs)

The 2017 treatment guidelines from the Veterans Health Administration and Department of Defense (VA/DoD) and the American Psychological Association (APA) offer recommendations for treating post-traumatic stress disorder (PTSD) [48,49]. These guidelines highly recommend three psychological therapies: prolonged exposure (PE), cognitive processing therapy (CPT), and trauma-focused cognitive behavioral therapy (CBT). All these therapies, which have substantial evidence backing their effectiveness, focus directly on the traumatic event’s memories, thoughts, and feelings [50].

In the area of psychotherapeutic treatments for significant trauma, CBT is particularly effective in addressing ingrained cognitive patterns that intensify emotional distress and maladaptive behavior [51]. Cognitive therapy is the cornerstone of the CBT approach, methodically guiding patients to recognize that beliefs developed in the context of severe trauma may be unfounded [52]. By illuminating potentially distorted perceptions, therapists help patients disentangle overwhelmingly negative interpretations of their experiences. At the same time, cognitive restructuring digs deeper into these maladaptive thought patterns, encouraging patients to challenge and reframe such thoughts [53].

It is invaluable in altering tendencies to overgeneralize negative experiences and cultivating healthier, more balanced understandings of traumatic events [54]. As reported by Bisson et al. [55], CBT for post-traumatic stress disorder (PTSD) showed similar efficacy to eye movement desensitization and reprocessing (EMDR) [56,57]. Both treatments outperformed conventional therapies, medication treatment, and alternative treatments such as supportive counseling for PTSD [58,59]. Nevertheless, the effectiveness of eye movement desensitization therapy remains controversial.

More research is needed to understand the cognitive processing of traumatic events, especially given the limitations of current diagnostic models such as the DSM-III-R. These models often fail to account for the subjective nature of trauma, leading to potential misdiagnosis and negatively impacting recovery. This problem is particularly pronounced in professions with frequent exposure to trauma, such as emergency services, where the cumulative effect of traumatic experiences presents unique diagnostic and treatment challenges (Parrot &Howes, 1991) [59].

In addition, there is a critical need for research focused on prevention, especially for individuals in high-risk roles. This would include exploring cognitive strategies to better prepare for and adapt to traumatic situations, including modifying perceptions of invulnerability and personal responsibility. Such research could lead to more effective intervention strategies, thereby improving outcomes for those who are regularly exposed to traumatic events [60].

### 3.9. Exposure Therapy

One of the most prominent therapies for the treatment of PTSD, prolonged exposure (PE), was developed by Dr. Edna Foa [61] and involves systematic exposure to traumatic memories (imaginal exposure) or triggers (in vivo exposure) without avoidance behavior. This technique provides an objective way to deal with traumatic experiences and is shown to significantly reduce PTSD symptoms. This treatment is designed to help patients directly confront and methodically process their traumatic memories. Although intense, it has proven effective, especially when customized to an individual’s readiness and resilience. Exposure therapy for massive trauma involves a gradual and controlled approach to confronting traumatic memories or stimuli [62]. The process typically begins with establishing a strong therapeutic alliance and teaching coping skills to manage anxiety. Once the basics are established, the patient is guided through repeated reliving or revisiting of the traumatic event in a safe and supportive environment, either through imaginal exposure—mentally reliving the event—or in vivo exposure—approaching situations or places associated with the trauma. This systematic desensitization helps to reduce the power of the traumatic memory and the avoidance behaviors that often accompany it. However, because of its inherently confrontational nature, exposure therapy must be approached with caution and is not always the first choice of treatment for those dealing with the aftermath of massive trauma. It is critical that therapists assess the potential for re-traumatization and ensure that the patient is not overwhelmed by the process. Careful implementation of exposure therapy as part of a broader CBT strategy is underscored by research confirming its effectiveness in addressing the deep and multifaceted aftermath of severe trauma, particularly in conditions such as post-traumatic stress disorder (PTSD) and acute stress disorder (ASD) [63]. Another kind of cognitive therapy combining exposure techniques with cognitive therapy components is cognitive processing therapy (CPT) for PTSD, which addresses conflicting beliefs and meanings of traumatic events, as well as future expectations. It involves creating a detailed narrative of the trauma that is reviewed daily. Studies show that CPT is effective and compares well with prolonged exposure therapy, but it is unclear whether its effectiveness would hold if the exposure elements were omitted [59].

### 3.10. Psychoeducation

Psychoeducation within cognitive behavioral therapy (CBT) serves primarily as a cornerstone for empowering patients who have experienced massive trauma. It provides critical knowledge for understanding trauma-related symptoms and equips them to effectively navigate and manage stress reactions [64]. In early CBT interventions, anxiety management skills are emphasized, including progressive muscle relaxation and controlled breathing exercises [65]. Psychoeducation is essential in helping patients manage acute distress, a common response to trauma. This method helps in educating patients about the physical, emotional, and cognitive responses to trauma, thereby allowing survivors to place their symptoms in a broader neurobiological context. This knowledge enables survivors to see their heightened vigilance, avoidance, or dissociation not as personal failings, but as part of the brain’s natural protective mechanisms. This reframing can reduce feelings of guilt and confusion, foster a sense of self-compassion, and reduce the isolation that often exacerbates traumatic experiences. Understanding the adaptive nature of these responses also helps individuals develop a more empathetic self-perception and promotes resilience, which is essential to their journey to recovery [66].

### 3.11. Mindfulness

Mindfulness is a therapeutic practice that focuses on present-moment awareness and can act as an anchor, allowing individuals to disengage from stressful memories and remain grounded in the present. Two notable approaches that incorporate mindfulness are mindfulness-based stress reduction (MBSR) and mindfulness-based cognitive therapy (MBCT) [67]. MBSR is typically an eight-week program that integrates meditation practices and psychoeducation, teaching individuals to cultivate awareness of their bodily sensations, feelings, and thoughts. MBCT, on the other hand, adds mindfulness exercises to traditional cognitive behavior strategies to help people identify and break the cycle of chronic negative thought patterns.

### 3.12. Social Skills Training

As survivors often struggle with issues of trust, emotional expression, and heightened responsiveness to social cues, interpersonal dynamics can become distorted after a traumatic experience [68]. Aiming to restore balance in this regard is social skills training, a methodical therapeutic approach [69].

Social skills training (SST) [70] is a systematic therapy that uses role-playing, feedback, and guided interaction to improve communication, assertiveness, and social cognition [71]. The goal of this training program is to provide participants with the tools they need to effectively communicate, assert themselves, and interpret and respond to social signals in order to increase their confidence in social situations and enhance the quality of their relationships [71,72,73].

### 3.13. Group Therapy 

Group therapy is a crucial treatment method for individuals who have experienced significant trauma, which can lead to deep-seated feelings of isolation. As a result, group therapy is a highly effective therapeutic method [74]. In a group setting, survivors can find comfort in knowing that they are not alone in their experiences, share stories, exchange coping techniques, and draw strength from the resilience of the community [75]. Moreover, observing the progress and recovery of peers can inspire hope, reinforcing the belief that healing is achievable.

Engaging with other individuals who have endured similar profound traumas provides a distinct, shared understanding, fostering a sense of community and lessening feelings of isolation [76]. These sessions are structured to provide safety and support, empowering participants to explore their traumatic experiences while establishing a connection within a collective environment. When survivors interact with each other, they can learn new coping mechanisms and adopt different perspectives, which can lead to corrective emotional experiences. The effectiveness of therapy comes not only from the shared stories but also from the observation of resilience, which is essential to the recovery process [77]. Despite the known benefits, research on the nuances and consequences of group therapy for mass trauma is limited, underscoring the need for broader studies.

Overall, the treatment of mass trauma requires a multifaceted approach in recognition of the profound uniqueness of the personal experience and the healing journey. Rather, an intelligent blend of evidence-based interventions, deeply rooted in an understanding of the trauma history of the victim, holds the promise of true recovery. Tailoring interventions to each individual’s unique needs and resiliency patterns is critical so that survivors can acquire cognitive and emotional tools to help them reclaim their lives and cope with the multiple challenges arising from their traumatic experiences.

### 3.14. Pharmacotherapy

Pharmacological interventions may be needed to accompany therapeutic efforts in the psychological scars of trauma [78]. Certain medications have been shown to be effective in alleviating mood and anxiety disorders resulting from trauma, particularly selective serotonin reuptake inhibitors (SSRIs) [79]. Depending on individual symptoms, other types of medications, such as benzodiazepines or antipsychotics, may also be used. To ensure optimal outcomes and minimize potential side effects, it is essential that a psychiatrist closely monitors any pharmacotherapy regimen [80].

## 4. Discussion

Severe trauma produces a variety of lasting neuropsychological effects [8,10], particularly when it results in post-traumatic stress disorder (PTSD). Individuals affected by trauma often exhibit deficits in basic cognitive functions, including attention and working memory. This may result in impairments in routine task performance and decision-making. PTSD also significantly impairs executive functioning, affecting critical cognitive aspects such as flexibility and regulation. As a result of the effects of severe trauma, individuals may have difficulty rapidly adapting to new environments and maintaining cognitive coherence [81,82,83]. In addition, individuals who have experienced severe trauma may exhibit a cognitive processing delay compared to their unaffected counterparts [16,17] as a result of a reduced rate of information processing, which is an often-overlooked consequence. These neuropsychological implications illustrate that the debilitating effects of severe trauma extend beyond emotional and psychological distress to cognitive functioning, exacerbating difficulties in recovery and everyday adjustment [84,85]. The following are critical observations that can be made:

The interventions presented here need to be evaluated critically, considering the deep-rooted neurobiological effects of trauma on individuals. Designing effective trauma interventions requires a multifaceted approach beyond simple recognition of an individual’s specific trauma narrative [86]. By integrating neuropsychological findings with a personalized, narrative-based approach to the brain–mind social environment interaction, interventions can be precisely customized to support recovery, allowing for a more holistic and effective response to the multifaceted nature of trauma [87,88,89]. However, such interventions require a comprehensive understanding of neuropsychological issues, particularly how trauma affects brain structure and function. Trauma can imprint on the brain in complex ways, affecting memory, concentration, emotional regulation, and threat perception [11], and it is essential that both aspects be addressed for successful interventions, given that clinicians are aware of recent research on brain plasticity, memory, and the neurobiological pathways of stress and recovery [90]. Therefore, effective interventions must address the individual’s personal experience, validating and working within the context of their lived trauma, while integrating strategies based on insights into the neuropsychological changes induced by trauma.

Recent research indicates that cognitive behavioral therapy (CBT) is a reliable method for treating post-traumatic stress disorder (PTSD), providing immediate and long-term relief for various types of traumas [91,92,93]. CBT proves to be as effective as, or more effective than, alternative psychological treatments and/or medication, even via digital platforms providing CBT [94]. Progressive muscle relaxation and controlled breathing are effective therapeutic practices and essential life skills that assist patients with regulating stress responses, creating a sense of control and increasing resilience [95]. Research strongly suggests that the benefits of CBT are based on neurological and psychological changes [96]. Additionally, CBT has been found to be valuable for treating trauma in preschool-aged children and adolescents, yielding positive outcomes. Furthermore, it has been adjusted to suit various cultures and is efficiently administered by community-based therapists with brief training in both individual and group settings, and has been proven effective for trauma after events such as catastrophic earthquakes [97]. Despite the plethora of studies that report success with cognitive behavioral therapy (CBT), up to 50% of individuals may not respond to it [98]. This can likely be attributed to a variety of factors, such as co-occurring disorders and certain sample characteristics. Although CBT is accepted for preventing the onset of PTSD, particularly when administered early to those at high risk, research is still inadequate to create a comprehensive framework for guidelines. Particularly in developing countries affected by large-scale disasters, further research is needed for the benefit of public health [99].

Exposure therapy is a validated treatment for PTSD and other trauma-related disorders with significant empirical support. The effectiveness of prolonged exposure (PE) is widely recognized, and valuable resources, such as Foa, Hembree, and Rothbaum’s Therapist’s Guide, provide a thorough overview for healthcare professionals [100]. Another pioneering approach is virtual reality exposure therapy (VRET), which uses a virtual environment simulation to immerse PTSD patients, particularly veterans, in their traumatic experiences and is particularly effective in military mental health [101,102,103,104]. Studies by Wald and Taylor [105] demonstrated the effectiveness of anxiety management techniques in the context of exposure therapy in alleviating acute stress symptoms and PTSD. These studies confirm the critical role of exposure therapy in the treatment of trauma by demonstrating its flexibility and therapeutic efficacy.

Research suggests that incorporating psychoeducation into therapeutic interventions can be beneficial for individuals who have experienced trauma [106,107]. Specifically, psychoeducation aims to explain common reactions to trauma and can increase understanding and reduce blame and isolation, emphasizing the importance of psychoeducation in helping patients understand normal reactions to trauma and facilitating their recovery process [108].

Mindfulness can be beneficial for many mental health conditions, but it raises unique considerations for those suffering from trauma. Practicing mindfulness can inadvertently trigger the emergence of intrusive memories, as the practice promotes an awareness that can bring past traumas into the present moment [67]. This paradox is highlighted in research such as the study by Kearney et al., which suggests that although mindfulness-based stress reduction (MBSR) may improve PTSD symptoms, it may initially increase distress due to the confrontation of traumatic memories [109]. Similarly, dialectical behavior therapy (DBT), which has mindfulness as a core component, has shown potential in the treatment of complex trauma. Research by Harned et al. supports the efficacy of dialectical behavior therapy (DBT), even though participants may experience initial challenges with the mindfulness exercises [110]. This study suggests that mindfulness can serve as a valuable technique for individuals in trauma recovery; however, it is critical that mindfulness practices be implemented with appropriate support to address potential difficulties associated with the re-experiencing of traumatic events during practice.

In social skills training (SST), individuals are taught effective communication strategies, assertiveness skills, and techniques for interpreting and responding to social cues that can improve confidence in social situations and facilitate more meaningful relationships. Through role-playing, feedback sessions, and guided interactions, SST encourages participants to identify and challenge negative beliefs about themselves and others that may impede their ability to form healthy relationships [111,112,113]. Research has demonstrated the effectiveness of SST in various settings, including improving the social competence of individuals with schizophrenia, a group that shares similar social deficits with those who have survived trauma. Mueser and colleagues also found that SST improved social functioning and reduced symptoms of PTSD, suggesting a dual therapeutic benefit [114]. Assertiveness training, one of the key components of SST, has been shown by Heimberg and colleagues to increase social confidence in individuals with social anxiety, often associated with PTSD [115]. These cases support the use of SST as a means of promoting social trust, potentially enabling trauma survivors to develop more meaningful relationships and improve overall social integration [116].

In group therapy, individuals can share and process traumatic experiences in supportive settings [51]. Sharing traumatic experiences in a group modulates the neural pathways linked to these memories. Research such as that by Neuner and colleagues (2004) has demonstrated the effectiveness of narrative exposure in group settings for reducing symptoms of PTSD, suggesting that such therapeutic interventions can modify the distress associated with traumatic memories [74,111]. Further highlighting the therapeutic potential of group dynamics, research by Sloan et has highlighted the effectiveness of group cognitive behavioral therapy in reducing PTSD symptoms in combat veterans [117]. However, the potential for retraumatization within group settings, as discussed by Luxton et al. (2010), highlights the complexity of these interactions and emphasizes the need for careful guidance to ensure a safe and constructive experience for participants [103]. These studies confirm that group therapy can be critical in promoting resilience and recovery but requires careful attention to members’ individual and collective experiences to avoid adverse outcomes.

Pharmacotherapy, particularly with selective serotonin reuptake inhibitors (SSRIs), is significant in managing symptoms associated with trauma-related disorders [118]. SSRIs correct neurotransmitter imbalances, targeting primarily serotonin dysregulation, to alleviate symptoms such as anxiety and depression [119]. However, questions remain about the long-term effects of these drugs on the brain’s complex neurochemistry. While selective serotonin reuptake inhibitors (SSRIs) may alleviate symptoms in the short term, as noted in studies such as Hoskins et al.’s [120], a systematic review of their impact on neural pathway healing is controversial. Research suggests that long-term use of SSRIs may lead to adaptive changes in brain function that are not fully understood and may not always be beneficial [121] This underscores the importance of ongoing assessment and a balanced strategy regarding the administration of medications to treat trauma, considering both the benefits and the nuances of brain chemistry.

It is worth noting that, according to some studies, psychological interventions did not significantly improve neurocognitive functions, except for memory, in research primarily focused on trauma treatment rather than direct cognitive improvement [122]. Cognitive training focusing on specific neurocognitive aspects also showed conflicting results in improving these functions [123]. However, combining treatments such as CBT with pharmacotherapy seems to be more effective in improving memory in PTSD patients, with paroxetine showing promise in improving verbal memory, suggesting a need for further research.

Last but not least, interventions addressing the consequences of severe trauma must consider cultural and individual differences recognizing that the impact of trauma is experienced through the lens of cultural background, personal history, and social context [9]. Cultural diversity in interventions requires recognition and consideration of the beliefs, values, norms, and practices that shape an individual’s or community’s response to trauma. Being culturally competent is therefore essential to ensure that support mechanisms are appropriate and respectful, thereby increasing their effectiveness and the likelihood of positive outcomes. Individual differentiation requires an understanding of the different ways in which individuals experience and process traumatic events [124]. Personal coping mechanisms, resilience, and psychological profiles vary widely, and interventions must adapt flexibly to these differences [125,126].

In practice, culturally familiar healing practices, language-appropriate services, and community outreach can be used to implement interventions that consider both cultural and individual differences. Culturally competent care is also a requirement that honors and understands the individual’s cultural background, as cultural factors can significantly influence the experience and expression of trauma.

Furthermore, adopting a public health approach to trauma and traumatic stress involves a significant shift in focus for trauma professionals and organizations like the International Society for Traumatic Stress Studies (ISTSS) and the European Society for Traumatic Stress Studies (ESTSS) [127]. This approach emphasizes the need for prevention strategies to mitigate the impact of trauma on individuals and society. By integrating preventive measures, these professionals and societies can broaden their societal impact and contribute more effectively to addressing trauma-related challenges [60].

In conclusion, the effectiveness of interventions for trauma must be objectively assessed, especially considering the complex neuropsychological consequences of trauma that affect both psychological and neurological issues. Ultimately, the successful management of trauma, especially in a clinical environment, requires thorough and consistent assessment, precise terminology, and balanced viewpoints.

## 5. Limitations

This narrative literature review is subject to the following methodological limitations and inherent complexities of synthesizing diverse studies on a multifaceted issue.

Firstly, the scope and quality of existing studies significantly influence the review’s comprehensiveness and reliability; gaps in research or methodological flaws in the original studies can skew the findings. The rapidly evolving nature of neuropsychology and trauma interventions means that such reviews can quickly become outdated. Additionally, the subjective nature of study selection and interpretation can introduce bias. The generalizability of findings is often limited, especially when the reviewed studies focus on specific populations. As review articles synthesize existing studies rather than present new empirical data, they may overlook areas where data are sparse. Cultural and contextual differences in neuropsychological impacts and intervention effectiveness, which are often not fully addressed, further limit the review’s applicability. The variability in interventions and their effectiveness, along with ethical and practical considerations in trauma research, add to these constraints. Lastly, the interdisciplinary nature of studying massive trauma and its neuropsychological consequences may not be adequately represented, potentially overlooking critical insights from various relevant fields. Understanding these limitations is essential for a nuanced interpretation of the review’s findings.

## 6. Conclusions

The profound neuropsychological effects of trauma underscore the complex relationship between experience and brain function, affecting key brain regions and disrupting neurochemical balance, leading to challenges and opportunities. The inherent neuroplasticity of the brain offers a promising pathway to recovery and underscores the importance of evidence-based interventions that are delivered in a timely manner. This research will not only reveal the complexity of the human brain but also provide guidelines for achieving recovery and resilience in challenging situations of massive trauma.

## References

[1] Naushad V.A., Bierens J.J., Nishan K.P., Firjeeth C.P., Mohammad O.H., Maliyakkal A.M., ChaliHadan S., Schreiber M.D. (2019). A Systematic Review of the Impact of Disaster on the Mental Health of Medical Responders. Prehospital Disaster Med..

[2] Cherry K.E., Sampson L., Galea S., Marks L.D., Nezat P.F., Baudoin K.H., Lyon B.A. (2016). Optimism and Hope after Multiple Disasters: Relationships to Health-Related Quality of Life. J. Loss Trauma.

[3] Schwent Shultz L.A., Rose B., Fink J.W. (2012). Clinical Neuropsychology. Appl. Neuropsychol. Adult.

[4] Vasterling J.J., Grande L., Graefe A.C., Alvarez J.A. (2019). Neuropsychological Assessment of Posttraumatic Stress Disorder (PTSD). Handbook of Medical Neuropsychology.

[5] Al Jowf G.I., Ahmed Z.T., An N., Reijnders R.A., Ambrosino E., Rutten B.P.F., de Nijs L., Eijssen L.M.T. (2022). A Public Health Perspective of Post-Traumatic Stress Disorder. Int. J. Environ. Res. Public Health.

[6] Shalev A.Y., Tuval-Mashiach R., Hadar H. (2004). Posttraumatic stress disorder as a result of mass trauma. J. Clin. Psychiatry.

[7] Bonanno G.A., Brewin C.R., Kaniasty K., Greca A.M.L. (2010). Weighing the Costs of Disaster. Psychol. Sci. Public Interest.

[8] Li M., Leidner B., Hirschberger G., Park J. (2022). From Threat to Challenge: Understanding the Impact of Historical Collective Trauma on Contemporary Intergroup Conflict. Perspect. Psychol. Sci..

[9] Schnyder U., Bryant R.A., Ehlers A., Foa E.B., Hasan A., Mwiti G., Kristensen C.H., Neuner F., Oe M., Yule W. (2016). Culture-sensitive psychotraumatology. Eur. J. Psychotraumatol..

[10] Brenner L.A. (2011). Neuropsychological and neuroimaging findings in traumatic brain injury and post-traumatic stress disorder. Dialogues Clin. Neurosci..

[11] Kira I.A., Templin T., Lewandowski L., Ramaswamy V., Ozkan B., Abou-Mediane S., Mohanesh J., Alamia H. (2011). Cumulative Tertiary Appraisals of Traumatic Events Across Cultures: Two Studies. J. Loss Trauma.

[12] Somasundaram D.J., Sivayokan S. (1994). War Trauma in a Civilian Population. Br. J. Psychiatry.

[13] Bremner J.D. (2006). Traumatic stress: Effects on the brain. Dialogues Clin. Neurosci..

[14] McNally R. (2006). Cognitive abnormalities in post-traumatic stress disorder. Trends Cogn. Sci..

[15] van der Kolk B. (2000). Posttraumatic stress disorder and the nature of trauma. Dialogues Clin. Neurosci..

[16] McCabe D.P., Roediger H.L., McDaniel M.A., Balota D.A., Hambrick D.Z. (2010). The relationship between working memory capacity and executive functioning: Evidence for a common executive attention construct. Neuropsychology.

[17] Schuitevoerder S., Rosen J.W., Twamley E.W., Ayers C.R., Sones H., Lohr J.B., Goetter E.M., Fonzo G.A., Holloway K.J., Thorp S.R. (2013). A meta-analysis of cognitive functioning in older adults with, PTSD. J. Anxiety Disord..

[18] Scott J.C., Matt G.E., Wrocklage K.M., Crnich C., Jordan J., Southwick S.M., Krystal J.H., Schweinsburg B.C. (2015). A quantitative meta-analysis of neurocognitive functioning in posttraumatic stress disorder. Psychol. Bull..

[19] Aupperle R.L., Melrose A.J., Stein M.B., Paulus M.P. (2012). Executive function and PTSD: Disengaging from trauma. Neuropharmacology.

[20] Woon F.L., Farrer T.J., Braman C.R., Mabey J.K., Hedges D.W. (2016). A meta-analysis of the relationship between symptom severity of Posttraumatic Stress Disorder and executive function. Cogn. Neuropsychiatry.

[21] Bremner J.D. (2002). Does Stress Damage the Brain? Understanding Trauma-Related Disorders from a Mind-Body Perspective.

[22] Breiter H.C., Etcoff N.L., Whalen P.J., AKennedy W., Rauch S.L., Buckner R.L., Strauss M.M., EHyman S., Rosen B.R. (1996). Response and Habituation of the Human Amygdala during Visual Processing of Facial Expression. Neuron.

[23] Tull M.T., Vidaña A.G., Betts J.E. (2020). Emotion regulation difficulties in PTSD. Emotion in Posttraumatic Stress Disorder.

[24] Chesney S.A., Gordon N.S. (2016). Profiles of emotion regulation: Understanding regulatory patterns and the implications for posttraumatic stress. Cogn. Emot..

[25] Admon R., Lubin G., Stern O., Rosenberg K., Sela L., Ben-Ami H., Hendler T. (2009). Human vulnerability to stress depends on amygdala’s predisposition and hippocampal plasticity. Proc. Natl. Acad. Sci. USA.

[26] Noël X., Van Der Linden M., Bechara A. (2006). The neurocognitive mechanisms of decision-making, impulse control, and loss of willpower to resist drugs. Psychiatry.

[27] Kim S., Lee D. (2011). Prefrontal cortex and impulsive decision making. Biol. Psychiatry.

[28] Avramidou K., Theodoratou M. (2023). Psychological Consequences of COVID on General Population.

[29] Theodoratou M., Kougioumtzis G.A., Kaltsouda A., Katsarou D., Siouti Z., Sofologi M., Tsitsas G., Flora K. (2023). Neuropsychological Aspects and Interventions for Internet Addiction in Adolescents with Asperger’s Syndrome-Narrative Review. Neurol. Neurosci..

[30] Toth S.L., Cicchetti D. (1998). Remembering, forgetting, and the effects of trauma on memory: A developmental psychopathology perspective. Dev. Psychopathol..

[31] Iyadurai L., Visser R.M., Lau-Zhu A., Porcheret K., Horsch A., Holmes E.A., James E.L. (2019). Intrusive memories of trauma: A target for research bridging cognitive science and its clinical application. Clin. Psychol. Rev..

[32] Kessler H., Holmes E.A., Blackwell S.E., Schmidt A.C., Schweer J.M., Bücker A., Herpertz S., Axmacher N., Kehyayan A. (2018). Reducing intrusive memories of trauma using a visuospatial interference intervention with inpatients with posttraumatic stress disorder (PTSD). J. Consult. Clin. Psychol..

[33] Wilson B.A., Sohlberg M.M., Mateer C.A. (2001). Cognitive Rehabilitation. An Integrative Neuropsychological Approach.

[34] Sohlberg M.M., Mateer C.A. (1989). Introduction to Cognitive Rehabilitation: Theory and Practice.

[35] Bonsall M.B., Holmes E.A. (2023). Temporal dynamics of trauma memory persistence. J. R. Soc. Interface.

[36] Constantinidou F., Thomas R.D., Ashley M.J. (2010). Principles of cognitive rehabilitation in traumatic brain injury: An integrative neuroscience approach. Traumatic Brain Injury: Rehabilitation, Treatment, and Case Management.

[37] Badour C.L., Feldner M.T. (2013). Trauma-related reactivity and regulation of emotion: Associations with posttraumatic stress symptoms. J. Behav. Ther. Exp. Psychiatry.

[38] Lee K.H., Lee H.Y., Park I., Lee Y.J., Kim N., Jeon S., Kim S., Jeon J.E., Kim S.J. (2021). Neural correlates of emotional reactivity and regulation in traumatized North Korean refugees. Transl. Psychiatry.

[39] Theodoratou M., Kanellopoulou P., Nikitidis N., Farmakopoulou I. (2023). Coping strategies of Health Care Workers during third wave of COVID. Eur. Psychiatry.

[40] Theodoratou M., Gkintoni E., Farmakopoulou I. (2023). Executive Functions and Quality of Life in Neurodevelopmental Spectrum. Outline. Tech. Soc. Sci. J..

[41] Davidson R.J., McEwen B.S. (2012). Social influences on neuroplasticity: Stress and interventions to promote wellbeing. Nat Neurosci.

[42] Jak A.J., Crocker L.D., Aupperle R.L., Clausen A., Bomyea J. (2016). Neurocognition in PTSD: Treatment Insights and Implications. Behavioral Neurobiology of PTSD.

[43] Hyunnie A., Joo H.-S., Park C.-O. (2012). A review of trauma-related emotions: Shame, guilt, and anger. Korea J. Couns..

[44] Pierce Z.P., Johnson E.R., Kim I.A., Lear B.E., Mast A.M., Black J.M. (2023). Therapeutic interventions impact brain function and promote post-traumatic growth in adults living with post-traumatic stress disorder: A systematic review and meta-analysis of functional magnetic resonance imaging studies. Front. Psychol..

[45] Bisson J.I., Ehlers A., Matthews R., Pilling S., Richards D., Turner S. (2007). Psychological treatments for chronic post-traumatic stress disorder. Br. J. Psychiatry.

[46] Center for Substance Abuse Treatment (US) (2014). Chapter 3, Understanding the Impact of Trauma. Trauma-Informed Care in Behavioral Health Services.

[47] Hammond D.C. (2005). Neurofeedback treatment of depression and anxiety. J. Adult Dev..

[48] American Psychological Association (2017). Clinical Practice Guideline for the Treatment of Posttraumatic Stress Disorder (PTSD) in Adults.

[49] VA/DoD Clinical Practice Guideline Working Group (2017). VA/DoD Clinical Practice Guideline for the Management of Posttraumatic Stress Disorder and Acute Stress Disorder.

[50] Watkins L.E., Sprang K.R., Rothbaum B.O. (2018). Treating PTSD: A Review of Evidence-Based Psychotherapy Interventions. Front. Behav. Neurosci..

[51] Hofmann S.G., Asnaani A., Vonk I.J.J., Sawyer A.T., Fang A. (2012). The Efficacy of Cognitive Behavioral Therapy: A Review of Meta-analyses. Cogn. Ther. Res..

[52] Beck A.T. (1976). Cognitive Therapies and Emotional Disorders.

[53] Kliethermes M.D., Drewry K., Wamser-Nanney R. (2017). Trauma-Focused Cognitive Behavioral Therapy. Evidence-Based Treatments for Trauma Related Disorders in Children and Adolescents.

[54] Raja S., Hasnain M., Hoersch M., Gove-Yin S., Rajagopalan C. (2015). Trauma Informed Care in Medicine. Fam. Community Health.

[55] Bisson J., Andrew M. (2007). Psychological treatment of post-traumatic stress disorder (PTSD). Cochrane Database of Systematic Reviews [Internet].

[56] Shapiro F. (2001). Eye Movement Desensitization and Reprocessing: Basic Principles, Protocols, and Procedures.

[57] Wadji D.L., Martin-Soelch C., Camos V. (2022). Can working memory account for EMDR efficacy in PTSD?. BMC Psychol..

[58] Acarturk C., Konuk E., Cetinkaya M., Senay I., Sijbrandij M., Gulen B., Cuijpers P. (2016). The efficacy of eye movement desensitization and reprocessing for post-traumatic stress disorder and depression among Syrian refugees: Results of a randomized controlled trial. Psychol Med..

[59] Parrott C.A., Howes J.L. (1991). The Application of Cognitive Therapy to Posttraumatic Stress Disorder.

[60] Magruder K.M., Kassam-Adams N., Thoresen S., Olff M. (2016). Prevention and public health approaches to trauma and traumatic stress: A rationale and a call to action. Eur. J. Psychotraumatol..

[61] Foa E.B., Rothbaum B.O. (1989). Behavioural Psychotherapy for Post-traumatic Stress Disorder. Int. Rev. Psychiatry.

[62] Foa E., Hembree E., Rothbaum B.O. (2007). Prolonged Exposure Therapy for PTSD: Emotional Processing of Traumatic Experiences Therapist Guide.

[63] Beidel D.C., Frueh B.C., Neer S.M., Bowers C.A., Trachik B., Uhde T.W., Grubaugh A. (2019). Trauma management therapy with virtual-reality augmented exposure therapy for combat-related PTSD: A randomized controlled trial. J. Anxiety Disord..

[64] Brooks S.K., Weston D., Wessely S., Greenberg N. (2021). Effectiveness and acceptability of brief psychoeducational interventions after potentially traumatic events: A systematic review. Eur. J. Psychotraumatol..

[65] Hamdani S.U., Huma Z.E., Zafar S.W., Suleman N., Baneen U.U., Waqas A., Rahman A. (2022). Effectiveness of relaxation techniques ‘as an active ingredient of psychological interventions’ to reduce distress, anxiety and depression in adolescents: A systematic review and meta-analysis. Int. J. Ment. Health Syst..

[66] Elam T., Taku K. (2022). Differences Between Posttraumatic Growth and Resiliency: Their Distinctive Relationships with Empathy and Emotion Recognition Ability. Front. Psychol..

[67] de Vibe M., Bjørndal A., Tipton E., Hammerstrøm K., Kowalski K. (2012). Mindfulness Based Stress Reduction (MBSR) for Improving Health, Quality of Life, and Social Functioning in Adults. Campbell Syst. Rev..

[68] Markowitz J.C., Milrod B., Bleiberg K., Marshall R.D. (2009). Interpersonal Factors in Understanding and Treating Posttraumatic Stress Disorder. J. Psychiatr. Pract..

[69] Kaniasty K., Norris F. (2013). The experience of disaster: Individuals and communities sharing trauma. Response to Disaster.

[70] Tyler P.M., Aitken A.A., Ringle J.L., Stephenson J.M., Mason W.A. (2021). Evaluating social skills training for youth with trauma symptoms in residential programs. Psychol. Trauma: Theory Res. Pract. Policy.

[71] Kotijah S., Munfadlila A.W. (2019). Effectiveness of social skills training (SST) based on computer and manual for improving socialization and social function of scizofrenia patients: Systematic review. Int. J. Nurs. Midwifery Sci..

[72] Bellack A.S., Mueser K.T., Gingerich S., Agresta J. (2004). Social Skills Training for Schizophrenia: A Step-by-Step Guide.

[73] Martell C.R., Addis M.E., Jacobson N.S. (2001). Depression in Context: Strategies for Guided Action.

[74] Malhotra A., Baker J., Group Therapy (2022). NCBI Bookshelf. https://www.ncbi.nlm.nih.gov/books/NBK549812/.

[75] Schwartze D., Barkowski S., Strauss B., Knaevelsrud C., Rosendahl J. (2017). Efficacy of group psychotherapy for posttraumatic stress disorder: Systematic review and meta-analysis of randomized controlled trials. Psychother. Res..

[76] Yalom I.D., Leszcz M. (2005). The Theory and Practice of Group Psychotherapy.

[77] Stige S.H., Rosenvinge J.H., Træen B. (2013). A meaningful struggle: Trauma clients’ experiences with an inclusive stabilization group approach. Psychother. Res..

[78] Davidson J. (1992). Drug Therapy of Post-traumatic Stress Disorder. Br. J. Psychiatry.

[79] Vermetten E., Vythilingan M., Southwick S., Charney D., Bremner J. (2003). Long term treatment with paroxetine increae verbal declarative memory and hippocampal volume in PTSD.pdf. Biol. Psychiatry.

[80] Stahl S.M. (2013). Stahl’s Essential Psychopharmacology: Neuroscientific Basis and Practical Applications.

[81] Rosenheck R. (1986). Stress Response Syndromes. Psychiatr. Serv..

[82] Fisher S. (1993). Coping with Trauma. By Rolf Kleber and Danny Brom in Collaboration with Peter B. Defares Amsterdam/Lisse: Swets & Zeitlinger 1992. Br. J. Psychiatry.

[83] Brewin C.R., Gregory J.D., Lipton M., Burgess N. (2010). Intrusive images in psychological disorders: Characteristics, neural mechanisms, and treatment implications. Psychol. Rev..

[84] Kabat Zinn J. (1990). Full Catastrophe Living: Using the Wisdom of Your Body and Mind to Face Stress, Pain, and Illness.

[85] McFarlane A.C., Van Hooff M. (2009). Impact of childhood exposure to a natural disaster on adult mental health: 20year longitudinal followup study. Br. J. Psychiatry.

[86] Dass-Brailsford P. (2007). Practical Approach to Trauma: Empowering Interventions.

[87] LaCasse M. (2017). Rewriting the Narrativewith Logotherapy: Review of Man’s Searchfor Meaning. Am. J. Psychiatry Resid. J..

[88] Shalev A.Y., Peri T., Canetti L., Schreiber S. (1996). Predictors of PTSD in injured trauma survivors: A prospective study. Am. J. Psychiatry.

[89] Cramer S.C., Sur M., Dobkin B.H., O’Brien C., Sanger T.D., Trojanowski J.Q., Rumsey J.M., Hicks R., Cameron J., Chen D. (2011). Harnessing neuroplasticity for clinical applications. Brain.

[90] Kar N. (2011). Cognitive behavioral therapy for the treatment of post-traumatic stress disorder: A review. Neuropsychiatr. Dis. Treat..

[91] Scheeringa M.S., Salloum A., Arnberger R.A., Weems C.F., Amaya-Jackson L., Cohen J.A. (2007). Feasibility and effectiveness of cognitive–behavioral therapy for posttraumatic stress disorder in preschool children: Two case reports. J. Trauma. Stress.

[92] Mendes D.D., Mello M.F., Ventura P., De Medeiros Passarela C., De Jesus Mari J. (2008). A Systematic Review on the Effectiveness of Cognitive Behavioral Therapy for Posttraumatic Stress Disorder. Int. J. Psychiatry Med..

[93] Thew G.R., Rozental A., Hadjistavropoulos H.D. (2022). Advances in digital CBT: Where are we now, and where next?. Cogn. Behav. Therap..

[94] Pathak P., Mahal R., Kohli A., Nimbran V. (2013). Progressive Muscle Relaxation: An adjuvant therapy for reducing pain and fatigue among hospitalized cancer patients’ receiving radiotherapy. Int. J. Adv. Nurs. Stud..

[95] Månsson K.N.T., Lueken U., Frick A. (2021). Enriching CBT by Neuroscience: Novel Avenues to Achieve Personalized Treatments. J. Cogn. Ther..

[96] Beiva-Bianchi M., Cornejo F., Fresno A., Rojas C., Serrano C. (2018). Effectiveness of cognitive-behavioural therapy for post-disaster distress in post-traumatic stress symptoms after Chilean earthquake and tsunami. Gac. Sanit..

[97] Schwartzmann B., Quilty L.C., Dhami P., Uher R., Allen T.A., Kloiber S., Lam R.W., Frey B.N., Milev R., Müller D.J. (2023). Resting-state EEG delta and alpha power predict response to cognitive behavioral therapy in depression: A Canadian biomarker integration network for depression study. Sci. Rep..

[98] Mello P.G., Silva G.R., Donat J.C., Kristensen C.H. (2013). An Update on the Efficacy of Cognitive-Behavioral Therapy, Cognitive Therapy, and Exposure Therapy for Posttraumatic Stress Disorder. Int. J. Psychiatry Med..

[99] Hembree E.A., Rauch S.A.M., Foa E.B. (2019). Beyond the manual: The insider’s guide to Powers MB, Rothbaum BO. Recent advances in virtual reality therapy for anxiety and related disorders: Introduction to the special issue. J. Anxiety Disord..

[100] Foa E.B., Kozak M.J. (1986). Emotional processing of fear: Exposure to corrective information. Psychol. Bull..

[101] Neuner F., Schauer M., Klaschik C., Karunakara U., Elbert T. (2004). A Comparison of Narrative Exposure Therapy, Supportive Counseling, and Psychoeducation for Treating Posttraumatic Stress Disorder in an African Refugee Settlement. J. Consult. Clin. Psychol..

[102] Booysen D.D., Kagee A. (2021). Preliminary Effectiveness of Brief Prolonged Exposure Therapy for PTSD: Expanding Access to Effective Therapies. Clin. Case Stud..

[103] Luxton D.D., Skopp N.A., Maguen S. (2010). Gender differences in depression and PTSD symptoms following combat exposure. Depress. Anxiety.

[104] Carl E., Stein A.T., Levihn-Coon A., Pogue J.R., Rothbaum B., Emmelkamp P., Asmundson G.J.G., Carlbring P., Powers M.B. (2019). Virtual reality exposure therapy for anxiety and related disorders: A meta-analysis of randomized controlled trials. J. Anxiety Disord..

[105] Wald J., Taylor S. (2005). Interoceptive Exposure Therapy Combined with Trauma-related Exposure Therapy for Post-traumatic Stress Disorder: A Case Report. Cogn. Behav. Ther..

[106] Lee S.C., Rawlings M.A. (2023). Healing from trauma through psychoeducation: Understanding young adult client group experiences. Soc. Work. Groups.

[107] Whitworth J.D. (2016). The Role of Psychoeducation in Trauma Recovery: Recommendations for Content and Delivery. J. Evid.-Inf. Soc. Work.

[108] Schrader C., Ross A. (2021). A Review of PTSD and Current Treatment Strategies. Mo Med..

[109] Kearney D.J., McDermott K., Malte C., Martinez M., Simpson T.L. (2012). Effects of Participation in a Mindfulness Program for Veterans With Posttraumatic Stress Disorder: A Randomized Controlled Pilot Study. J. Clin. Psychol..

[110] Harned M.S., Korslund K.E., Foa E.B., Linehan M.M. (2012). Treating PTSD in suicidal and self-injuring women with borderline personality disorder: Development and preliminary evaluation of a Dialectical Behavior Therapy Prolonged Exposure Protocol. Behav. Res. Ther..

[111] Biglan A., Hayes S.C., Pistorello J. (2008). Acceptance and Commitment: Implications for Prevention Science. Prev. Sci..

[112] John C. (2000). Social skills training for schizophrenia: A step by step guide; Alan S. Bellack, Kim T. Meuser, Susan Gingerich and Julie Agresta, Guilford Press, New York (1997), xiv+288 pp, $30.00 (hardback). Behav. Res. Ther..

[113] Rus-Calafell M., Gutiérrez-Maldonado J., Ribas-Sabaté J. (2014). A virtual reality-integrated program for improving social skills in patients with schizophrenia: A pilot study. J. Behav. Ther. Exp. Psychiatry.

[114] Mueser K.T., Rosenberg S.D., Xie H., Jankowski M.K., Bolton E.E., Lu W., Hamblen J.L., Rosenberg H.J., McHugo G.J., Wolfe R. (2008). A randomized controlled trial of cognitive-behavioral treatment for posttraumatic stress disorder in severe mental illness. J. Consult. Clin. Psychol..

[115] Heimberg R.G., Dodge C.S., Hope D.A., Kennedy C.R., Zollo L.J., Becker R.E. (1990). Cognitive behavioral group treatment for social phobia: Comparison with a credible placebo control. Cogn. Ther. Res..

[116] Eslinger P.J., Anders S., Ballarini T., Boutros S., Krach S., Mayer A.V., Moll J., Newton T.L., Schroeter M.L., de Oliveira-Souza R. (2021). The neuroscience of social feelings: Mechanisms of adaptive social functioning. Neurosci. Biobehav. Rev..

[117] Sloan D.M., Feinstein B.A., Gallagher M.W., Beck J.G., Keane T.M. (2013). Efficacy of group treatment for posttraumatic stress disorder symptoms: A meta-analysis. Psychol. Trauma: Theory Res. Pract. Policy.

[118] Chu A., Wadhwa R. (2023). Selective Serotonin Reuptake Inhibitors. NCBI Bookshelf. https://www.ncbi.nlm.nih.gov/books/NBK554406/.

[119] Inoue T. (2018). Neuroscientific Understanding of the Mechanism of Action of SSRI in the Treatment of Anxiety Disorders. Anxiety Disord. Res..

[120] Hoskins M., Pearce J., Bethell A., Dankova L., Barbui C., Tol W.A., van Ommeren M., de Jong J., Seedat S., Chen H. (2015). Pharmacotherapy for post-traumatic stress disorder: Systematic review and meta-analysis. Br. J. Psychiatry.

[121] Rawson S. (2020). Trauma-Sensitive Schools. Applying Trauma-Sensitive Practices in School Counseling.

[122] Susanty E., Sijbrandij M., van Dijk W., Srisayekti W., de Vries R., Huizink A.C. (2022). The effects of psychological interventions on neurocognitive functioning in posttraumatic stress disorder: A systematic review. Eur. J. Psychotraumatol..

[123] Knotek S.E. (2012). Utilizing culturally responsive consultation to support innovation implementation in a rural school. Consult. Psychol. J. Pract. Res..

[124] Bedard-Gilligan M., Jaeger J., Echiverri-Cohen A., Zoellner L.A. (2012). Individual differences in trauma disclosure. J. Behav. Ther. Exp. Psychiatry.

[125] Fani N., Kitayama N., Ashraf A., Reed L., Afzal N., Jawed F. (2009). Neuropsychological functioning in patients with posttraumatic stress disorder following short-term paroxetine treatment. Psychopharmacol. Bull..

[126] Muniandy M., Richdale A.L., Lawson L.P. (2022). Coping-resilience profiles and experiences of stress in autistic adults. Autism Res..

[127] Schnyder U., Ehlers A., Elbert T., Foa E.B., Gersons B.P., Resick P.A., Shapiro F., Cloitre M. (2015). Psychotherapies for PTSD: What do they have in common?. Eur. J. Psychotraumatol..

